# Role of microRNAs in skeletal muscle hypertrophy

**DOI:** 10.3389/fphys.2013.00408

**Published:** 2014-01-16

**Authors:** Keisuke Hitachi, Kunihiro Tsuchida

**Affiliations:** Division for Therapies against Intractable Diseases, Institute for Comprehensive Medical Science, Fujita Health UniversityToyoake, Japan

**Keywords:** insulin-like growth factor-1, myostatin, protein kinase B (Akt), Smad3, skeletal muscle hypertrophy

## Abstract

Skeletal muscle comprises approximately 40% of body weight, and is important for locomotion, as well as for metabolic homeostasis. Adult skeletal muscle mass is maintained by a fine balance between muscle protein synthesis and degradation. In response to cytokines, nutrients, and mechanical stimuli, skeletal muscle mass is increased (hypertrophy), whereas skeletal muscle mass is decreased (atrophy) in a variety of conditions, including cancer cachexia, starvation, immobilization, aging, and neuromuscular disorders. Recent studies have determined two important signaling pathways involved in skeletal muscle mass. The insulin-like growth factor-1 (IGF-1)/Akt pathway increases skeletal muscle mass via stimulation of protein synthesis and inhibition of protein degradation. By contrast, myostatin signaling negatively regulates skeletal muscle mass by reducing protein synthesis. In addition, the discovery of microRNAs as novel regulators of gene expression has provided new insights into a multitude of biological processes, especially in skeletal muscle physiology. We summarize here the current knowledge of microRNAs in the regulation of skeletal muscle hypertrophy, focusing on the IGF-1/Akt pathway and myostatin signaling.

## Introduction

Skeletal muscle is the most abundant tissue in our body and is important in locomotion and metabolic adaptation. Adult skeletal muscle mass is mainly determined by a balance between muscle protein synthesis and degradation. Exercise, nutrients, and exogenous stimuli increase the rate of muscle protein synthesis and increase skeletal muscle mass (hypertrophy), whereas starvation, immobilization, aging, and diseases increase the rate of protein degradation and markedly reduce skeletal muscle mass (atrophy). In addition, some muscular disorders produce a larger but much weaker muscle (pseudo-hypertrophy) through degeneration and regeneration of myofibers (Tyler, 2003). Several signaling pathways are shown to be involved in the regulation of skeletal muscle mass. The insulin-like growth factor-1 (IGF-1)/Akt pathway and β-adrenergic pathway are positive regulators of skeletal muscle growth. In contrast, myostatin signaling, NF-κβ signaling, and glucocorticoid signaling negatively regulate skeletal muscle mass (Sandri, 2008).

MicroRNAs (miRNAs) are small non-coding RNAs that are highly conserved in eukaryotes. Currently, the important role of miRNAs is evident in diverse biological processes, including development, differentiation, homeostasis, and disease in vertebrate species (Sayed and Abdellatif, 2011). miRNAs function to fine tune gene expression by accelerating degradation of mRNA and/or by inhibiting translation (Bartel, 2009). Identification of muscle-specific miRNAs called myomiR (miR-1, miR-133a/b, miR-206, miR-208b, miR-499, and miR-486) has extended our knowledge of the molecular network in skeletal muscle. The myomiRs form a feedback regulatory loop with myogenic regulatory factors, such as MyoD and myogenin, to precisely regulate skeletal muscle plasticity. For example, miR-1, mir-27, miR-206, and miR-486 promote myoblast differentiation through the repression of Pax7 and Pax3 expression (Crist et al., 2009; Chen et al., 2010; Dey et al., 2011). Meanwhile, the expression of myomiRs is strictly controlled by MyoD, myogenin, MEF2, and Pax7 (Rao et al., 2006; Rosenberg et al., 2006; Liu et al., 2007; Dey et al., 2011).

**Figure 1:**
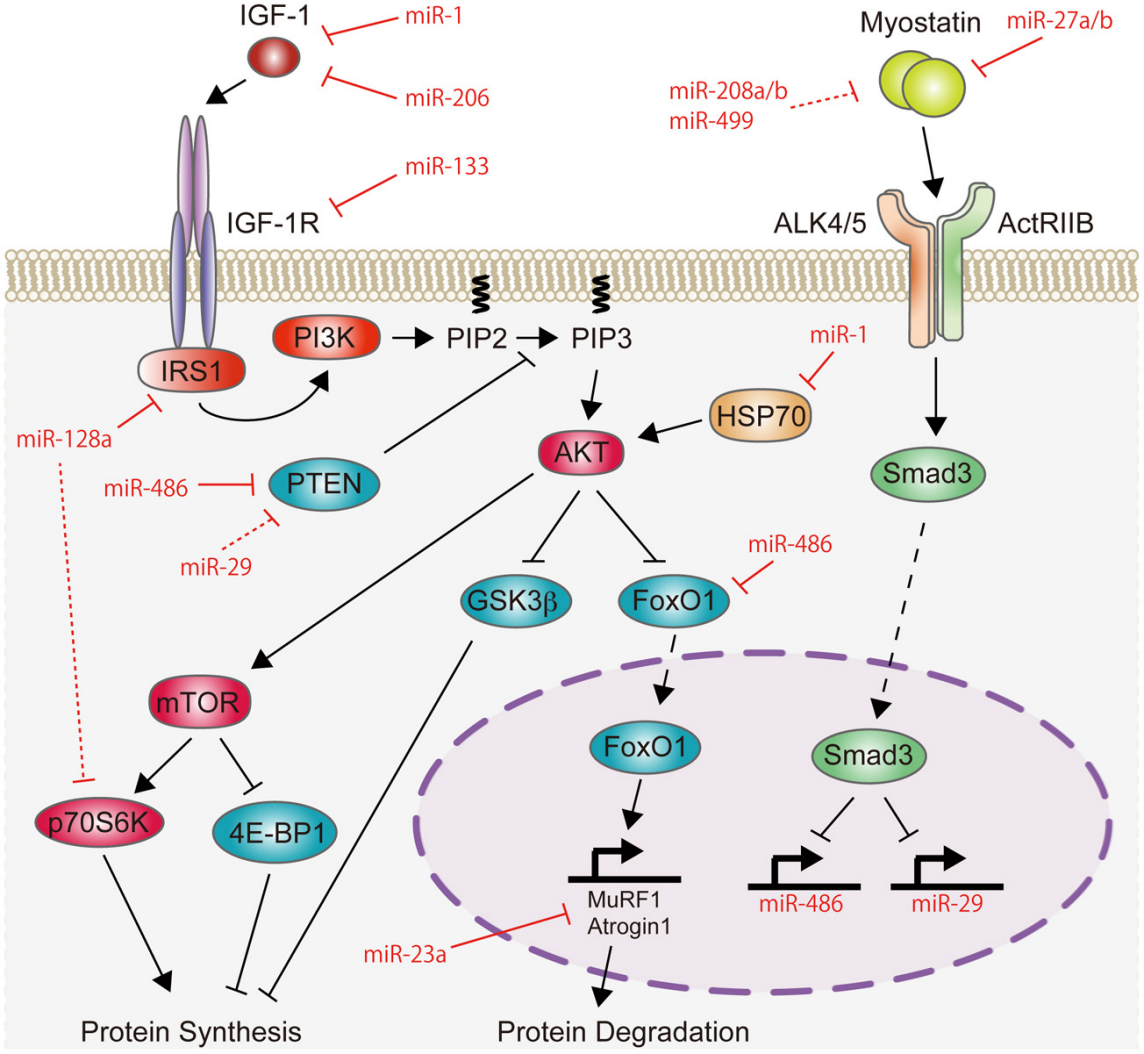
**Schematic representation of miRNAs involved in the regulation of the IGF-1/Akt pathway and myostatin signaling**. IGF-1 increases the activity of Akt protein. This activation of Akt is attenuated by PTEN. The activated state of Akt stimulates protein synthesis through mTOR. Akt also inhibits protein degradation by suppressing the activity of GSK3β and FoxO1, which induces protein degradation by activating the expression of MuRF1 and Atrogin1. Therefore, the IGF-1/Akt pathway induces skeletal muscle hypertrophy. Recent studies have shown that miR-1, miR-133, miR-206, and miR-128a negatively regulate the IGF-1/Akt pathway by targeting positive regulators of the IGF-1/Akt pathway (IGF-1, IGF-1R, IRS1, HSP70, or p70S6K), while miR-29, miR-486, and miR-23a positively regulate this pathway by targeting negative regulators (PTEN, FoxO1, MuRF1, or Atrogin1). In contrast to the IGF-1/Akt pathway, myostatin signaling is a negative regulator of skeletal muscle mass. Myostatin activates Smad3 protein and may inhibit protein synthesis by repressing the transcription of miR-486 and miR-29, which target PTEN and FoxO1 proteins. Conversely, myostatin expression is regulated by miR-27a/b, miR-208a/b, and miR-499. Red lines indicate the inhibitory function of miRNAs identified in skeletal muscle, while red-dashed lines represent the inhibitory function of miRNAs identified in cardiac muscle and cultured cells.

This mini-review highlights the recent findings of miRNAs in skeletal muscle hypertrophy, and focuses on the IGF-1/Akt pathway and myostatin signaling, because the fine balance between these two signaling pathways is essential for maintaining normal skeletal muscle mass (Schiaffino et al., 2013). The role of miRNAs on the IGF-1/Akt pathway and myostatin signaling is summarized in Figure 1. miRNAs also function in skeletal muscle atrophy and their role has been described in detail in recent reviews (Ma et al., 2012; Wang, 2013).

## The IGF-1/Akt pathway and microRNAs

IGF-1 binds to its own tyrosine kinase receptor IGF-1R and activates phosphatidylinositol-3-kinase (PI3K) through IRS1. Activated PI3K produces phosphatidylinositol-3,4,5,triphosphates (PIP3) and then induces activation of the Akt protein. Subsequently, Akt activates mammalian target of rapamycin (mTOR) and regulates down stream effector p70 ribosomal protein S6 kinase (p70S6K) and 4E-BP1, both of which control protein synthesis (Rommel et al., 2001). Akt also inactivates glycogen synthase kinase 3β (GSK3β), which blocks protein translation (Cross et al., 1995). In addition, Akt inhibits the nuclear translocation of FoxO family of transcription factors. Because FoxOs play a key role in the regulation of muscle atrophy-related genes, MuRF1, and Atrogin1 (Sandri et al., 2004), inactivation of FoxOs prevents muscle protein degradation. In this manner, the IGF-1/Akt pathway plays a central role in skeletal muscle hypertrophy. Indeed, ectopic Akt expression is sufficient for increasing the size of myofibers in adult mice (Bodine et al., 2001; Takahashi et al., 2002; Lai et al., 2004). Although the effect of IGF-1 on skeletal muscle mass mainly depends on development, IGF-1 can lead to increase skeletal muscle mass even in adulthood (Adams and McCue, 1998; Barton-Davis et al., 1998; Alzghoul et al., 2004; Schertzer et al., 2006).

Recent studies have identified several miRNAs that can modulate the muscle IGF-1/Akt pathway. In this section, we review the recently discovered miRNAs that target the IGF-1/Atk pathway in skeletal muscle.

### MicroRNA-1, -133, and -206

The expression levels of miR-1 and miR-133 are greatly increased during myogenesis (Ge and Chen, 2011), whereas the levels of their expression are reduced during skeletal muscle hypertrophy. Functional overload in mice decreases the levels of miR-1 and miR-133 expression with an increase in muscle weight (McCarthy and Esser, 2007). Resistance training with amino acid ingestion also decreases miR-1 expression in young men (Drummond et al., 2008). Overload and acute resistance exercise are shown to increase the levels of IGF-1 peptide in rats (Adams et al., 1999) and the activity of the Akt/mTOR in humans, respectively (Mayhew et al., 2009). Therefore, reduced miR-1 and miR-133 expression could contribute to the activation of the IGF-1/Akt pathway. Indeed, miR-1 and miR-133 inhibit the IGF-1/Akt pathway in C2C12 cells by targeting IGF-1, IGF-1R, and HSP70 (Elia et al., 2009; Huang et al., 2011; Kukreti et al., 2013), however, their targets in adult skeletal muscle remain to be defined.

Notably, inhibition of miR-206 in tilapia skeletal muscle promotes body growth with an increase in IGF-1 expression (Yan et al., 2013). However, although inhibition of miR-206 robustly increases C2C12 myotube width, gain and loss of function of miR-206 do not affect skeletal muscle size *in vivo* (Winbanks et al., 2013).

### MicroRNA-128a

To the best of our knowledge, miR-128a is the only miRNA that has the potential to increase skeletal muscle mass. In mice, miR-128a is highly expressed in brain and skeletal muscle (Lee et al., 2008), and its expression is increased during myoblast differentiation (Chen et al., 2006, 2010; Sun et al., 2010). Motohashi et al. (2013) identified IRS1 as a target of miR-128a in mice. They showed that overexpression of miR-128a repressed myoblast proliferation with a decrease in IRS1 protein and Akt activity. Interestingly, inhibition of miR-128a increased the size of C2C12 myotubes, with an increase in IRS1 protein and Akt activity. Notably, 4 weeks' administration of antisense miR-128 significantly induced skeletal muscle hypertrophy in mice. Given that miR-128 inhibits glioma tumor growth by targeting p70S6K (Shi et al., 2012), miR-128a may act as a negative regulator against multiple components of the IGF-1/Akt anabolic pathway.

### MicroRNA-486

An alternative promoter, located upstream of exon 39a of the *Ank1* gene, produces cardiac and skeletal muscle-specific Ank1.5 protein (also referred to as sAnk1), which connects sarcomeres to the sarcoplasmic reticulum (Zhou et al., 1997; Porter et al., 2005). miR-486 is transcribed from the final intron of the *Ank1.5* gene, and is co-expressed with *Ank1.5* mRNA in cardiac and skeletal muscles (Small et al., 2010).

miR-486 was initially identified as a downstream target of myocardin-related transcription factor-A in cardiomyocytes (Small et al., 2010). MyoD also directly stimulates the promoter activity of miR-486/*Ank1.5* and increases their expression during myoblast differentiation (Dey et al., 2011). miR-486 promotes the differentiation of satellite cells, adult skeletal muscle stem cells, by targeting *Pax7* mRNA (Dey et al., 2011), whereas muscle-specific transgenic overexpression of miR-486 shows delayed regeneration when skeletal muscle is injured (Alexander et al., 2011).

In addition, miR-486 is involved in the regulation of Akt activity by directly targeting the 3′ UTR of phosphatase and tensin homolog (PTEN) and FoxO1. Because PTEN inactivates the Akt protein (Stambolic et al., 1998), miR-486 increases the phosphorylation status of Akt with a decrease in PTEN and FoxO1 protein levels in mouse primary myotubes (Xu et al., 2012). miR-486 also decreases protein levels of PTEN, FoxO1, PDGFRβ, SFSR1, and SFSR3 in human myoblasts and myotubes (Alexander et al., 2011). We recently showed that miR-486 is necessary to maintain Akt activity in C2C12 myotubes. Furthermore, inhibition of miR-486 activity *in vivo* induced myofiber atrophy (Hitachi et al., 2014). Interestingly, ectopic miR-486 expression blocked the suppression of Akt activity caused by chronic kidney disease (CKD) and was sufficient for preventing skeletal muscle atrophy induced by CKD in mice (Xu et al., 2012). Therefore, miR-486 contributes to maintain skeletal muscle mass through the regulation of Akt activity.

## Myostatin signaling and microRNAs

Myostatin is a cytokine belonging to the transforming growth factor-β (TGF-β) superfamily. In contrast to the IGF-1/Akt pathway, myostatin signaling acts as a negative regulator of skeletal muscle mass. The importance of myostatin in skeletal muscle mass was first shown by McPherron et al. (1997). They generated mice with deletion of the *myostatin* gene, which resulted in doubled skeletal muscle weight due to an increase in muscle fiber size (hypertrophy) and increased muscle fiber number (hyperplasia). This groundbreaking research led to better understanding of the mechanism for excessive skeletal muscle growth observed in cattle, sheep, and dogs, which have naturally occurring mutations in the *myostatin* gene (Grobet et al., 1997; McPherron and Lee, 1997; Clop et al., 2006; Mosher et al., 2007). Importantly, a naturally occurring mutation in the *myostatin* gene was found in human with increased skeletal muscle mass (Schuelke et al., 2004). The importance of myostatin in muscle size was further supported by the finding that systemically administrated myostatin induces skeletal muscle atrophy in mice (Zimmers et al., 2002). Notably elevated myostatin expression is correlated with muscle wasting in cancer cachexia, heart failure, HIV, CKD, chronic obstructive pulmonary disease, and aging (Gonzalez-Cadavid et al., 1998; Yarasheski et al., 2002; Shyu et al., 2006; Costelli et al., 2008; Plant et al., 2010; Verzola et al., 2011). Currently, blockade of myostatin function is believed to be a promising therapy against muscle wasting caused by neuromuscular disorders, cancer, and aging (Han et al., 2013).

Myostatin signaling is transmitted intracellularly through serine/threonine kinase receptors, including activin receptor type IIB (ActRIIB) and activin receptor-like kinases (ALK4 or ALK5) (Rebbapragada et al., 2003). After binding of myostatin to its receptors, ALK4/ALK5 activates Smad2 and Smad3 by phosphorylation of the C-terminal domain (Langley et al., 2002; Rebbapragada et al., 2003). Phosphorylated Smad2/3 can form a complex together with Smad4, and it then moves to the nucleus to mediate transcriptional regulation of target genes. In early studies, satellite cells were once considered to be a major source of myostatin deficiency-induced muscular hypertrophy because myostatin inhibits satellite cell activation and self-renewal (McCroskery et al., 2003). Subsequently, several groups have shown a direct effect of myostatin on myofibrillar protein synthesis (Taylor et al., 2001; Welle et al., 2006, 2009, 2011). Recent studies have shown that satellite cells minimally contribute to skeletal muscle hypertrophy by inhibition of myostatin (Amthor et al., 2009; Lee et al., 2012; Wang and McPherron, 2012).

In 2006, a relationship between myostatin and miRNA was first shown in sheep. Clop et al. found a G to C single nucleotide polymorphism in the 3′ UTR of the *myostatin* gene in Texel sheep, which show remarkable skeletal muscle hypertrophy (Clop et al., 2006). This single nucleotide polymorphism changes the 3′ UTR of *myostatin* into the target of miR-206. Accordingly, the expression level of myostatin is low in Texel sheep. After this discovery, several miRNAs have been shown to be involved in myostatin signaling. In this section, we summarize the current knowledge of miRNAs related to myostatin signaling.

### MicroRNA-27

miR-27 consists of miR-27a and miR-27b, which differ by one nucleotide at position 19. During mice embryogenesis, miR-27b is expressed in the limb bud, tail bud, neural tube, heart, and somites (Crist et al., 2009). miR-27b expression is also upregulated during satellite cell differentiation in adult mice (Crist et al., 2009). Gain and loss of function of miR-27b initially indicated that miR-27b promotes myogenic differentiation by downregulating Pax3 expression (Crist et al., 2009). Consistent with this finding, miR-27b and *Pax3* mRNA are exclusively expressed in somites. miR-27b is expressed in myotomes and sclerotomes, while *Pax3* mRNA is expressed in dermomyotomes (Crist et al., 2009).

Skeletal muscle mainly consists of three types of fibers, which are slow oxidative, fast oxidative glycolytic, and fast glycolytic fibers. *Myostatin* mRNA is highly expressed in fast-twitch muscle, while its expression is low in slow-twitch muscle (Allen et al., 2008). Surprisingly, the expression level of *myostatin* pre-mRNA is indistinguishable between these two types of muscle. Allen and Loh resolved this paradox to discover post-transcriptional control of *myostatin* mRNA by miR-27. They showed that miR-27a/b is highly expressed in slow-twitch muscle and reduced the stability of *myostatin* mRNA by direct binding to the 3′ UTR (Allen and Loh, 2011). Therefore, low myostatin levels in slow-twitch muscle are maintained by miR-27 in a post-transcriptional mechanism. In mouse embryos at day 10.5, *myostatin* mRNA is expressed in the myotome compartment (McPherron et al., 1997) where miR-27b is expressed (Crist et al., 2009). Accordingly, the regulatory mechanism of *myostatin* mRNA may differ between adult and embryonic muscle.

Piedmontese cattle show the double-muscled phenotype, which is inherited, and is caused by a point mutation in the *myostatin* gene. Missense mutation of the *myostatin* gene G938A is translated to C313Y, and this alters the activity of myostatin protein (Berry et al., 2002), and induces muscle hypertrophy and hyperplasia (Kambadur et al., 1997; McPherron and Lee, 1997). Although 96% of Piedmontese cattle have a homozygosis mutation of the *myostatin* gene, different phenotypes of muscularity are observed in this breed. Greatly increased miR-27b expression with a decrease in myostatin expression have been found in Piedmontese cattle (Miretti et al., 2013). However, the cause of differential expression of miR-27b in this breed is unclear.

In humans, ingestion of leucine-enriched essential amino acids quickly stimulates muscle protein synthesis (Fujita et al., 2007) with a decrease in *myostatin* mRNA expression (Drummond et al., 2009). Expression levels of miR-27 have not yet been determined in this situation. However, considering the fact that treatment of 1 mM leucine decreases myostatin expression with an increase in miR-27a expression in C2C12 cells (Chen et al., 2013), miR-27 might contribute to reduce *myostatin* expression even in human skeletal muscle.

### MicroRNA-208a, -208b, and -499

Three related myomiRs, miR-208a, miR-208b, and miR-499, are encoded by introns of the *myosin* genes (miR-208a*/Myh6*, miR-208b*/Myh7*, and miR-499*/Myh7b*). Expression of miR-208a is heart specific, while miR-208b and miR-499 are expressed in cardiac and slow-twitch skeletal muscle (van Rooij et al., 2009). In skeletal muscle, miR-208b and miR-499 promote the formation of slow-twitch fibers, because these miRNAs increase the expression of slow muscle genes by repressing Sox6, Pur-β, and Sp3 (van Rooij et al., 2009). Accordingly, double knockout of miR-208b and miR-499 in mice leads to a loss of slow fibers in the soleus (van Rooij et al., 2009).

Recent studies have shown the direct association of miR-208a, miR-208b, and miR-499 with the 3′ UTR of *myostatin* by luciferase assays (Callis et al., 2009; Bell et al., 2010). Although miR-208a induces cardiac hypertrophy with decreased myostatin expression (Callis et al., 2009), whether miR-208b and miR-499 have potential to induce skeletal muscle hypertrophy has not been described. It is noteworthy that nutrient ingestion increases miR-208b and miR-499 expressions with a decrease in myostatin expression in human vastus lateralis muscle (Drummond et al., 2009). Therefore, future studies are required to determine the function of miR-208b and miR-499 in the regulation of skeletal muscle mass.

### MicroRNAs under the control of myostatin signaling

Thus far, we have described miRNAs that affect myostatin expression. Various studies have also investigated the opposite situation of whether myostatin-related signaling alters the expression of miRNAs. Davis et al. (2008) found that, in response to TGF-β and BMP, Smad proteins (Smad3 and Smad1/5) accelerated the processing of miR-21 in human smooth muscle cells. In this situation, Smad proteins interacted with RNA helicase p68, a component of the Drosha complex. Smad proteins were later shown to directly bind to the RNA-Smad binding element of primary transcripts of miRNAs and facilitate the interaction between miRNAs and the Drosha complex (Davis et al., 2010). BMP-2 is also likely to regulate the biogenesis of miR-206 using Smad proteins in C2C12 cells (Sato et al., 2009). Although altered expression of miR-1, miR-133a/b, and miR-206 is found in myostatin knockout mice (Rachagani et al., 2010), there is still no evidence that myostatin directly regulates miRNA processing.

miR-29 was initially shown to accelerate muscle differentiation by suppressing YY1 (Wang et al., 2008) and Akt3 (Wei et al., 2013). miR-29 also attenuates the inhibitory effect of TGF-β in muscle differentiation, and its expression is under regulation of TGF-β and Smad3 (Winbanks et al., 2011; Zhou et al., 2012). Interestingly, miR-29 activates the Akt pathway by targeting PTEN in cultured cells (Kong et al., 2011; Tumaneng et al., 2012). Goodman et al. (2013) recently showed that *in vivo* transfection of Smad3 reduced the Akt/mTOR activity and induced myofiber atrophy. In this situation, increased *PTEN* mRNA translation with a decrease in miR-29 promoter activity was observed. However, whether myostatin is involved in the regulation of miR-29 expression and the function of miR-29 in the regulation of skeletal muscle mass remain to be elucidated.

Most recently, we found greatly increased miR-486 expression in myostatin knockout mice. We showed that myostatin signaling repressed miR-486 expression at the transcriptional level using Smad3 protein (Hitachi et al., 2014). As described above, miR-486 increases the Akt activity by targeting PTEN, therefore, several miRNAs including miR-486 would contribute to induce skeletal muscle hypertrophy from inhibition of myostatin by activating the Akt pathway.

## Conclusion

Although miRNAs have been implicated in skeletal muscle development, regeneration, and function, our understanding of the molecular mechanisms underlying the regulation of skeletal muscle mass by miRNAs is still limited. Exercise, nutrition, and disease affect skeletal muscle mass postnatally, and they were also recently shown to alter the levels of skeletal muscle miRNA expression (Pasiakos and McClung, 2013; Zacharewicz et al., 2013). Therefore, to understand the exact role of miRNAs in skeletal muscle hypertrophy and atrophy, it is necessary to specifically investigate the molecular function of miRNAs in adult skeletal muscle without affecting development of skeletal muscle. Indeed, using electric pulse-mediated gene transfer in adult mice, Wada et al. (2011) clearly showed that ectopic expression of miR-23a was sufficient to prevent dexamethasone-induced skeletal muscle atrophy. Future studies using *in vivo* techniques, such as conditional transgenic and knockout technology, viral and electrophoretic transfer of DNA and RNA, or tail vein injection of nucleic acids with a modified backbone, will further define the exact role of miRNAs in the regulation of skeletal muscle mass.

### Conflict of interest statement

The authors declare that the research was conducted in the absence of any commercial or financial relationships that could be construed as a potential conflict of interest.
